# Establishment of a Comprehensive List of Candidate Antiaging Medicinal Herb Used in Korean Medicine by Text Mining of the Classical Korean Medical Literature, “*Dongeuibogam*,” and Preliminary Evaluation of the Antiaging Effects of These Herbs

**DOI:** 10.1155/2015/873185

**Published:** 2015-03-10

**Authors:** Moo Jin Choi, Byung Tae Choi, Hwa Kyoung Shin, Byung Cheul Shin, Yoo Kyoung Han, Jin Ung Baek

**Affiliations:** ^1^Division of Humanities and Social Medicine, School of Korean Medicine, Pusan National University, Yangsan 626-870, Republic of Korea; ^2^Division of Meridian and Structural Medicine, School of Korean Medicine, Pusan National University, Yangsan 626-870, Republic of Korea; ^3^Department of Korean Rehabilitation Medicine, Pusan National University Korean Medicine Hospital, Yangsan 626-789, Republic of Korea

## Abstract

The major objectives of this study were to provide a list of candidate antiaging medicinal herbs that have been widely utilized in Korean medicine and to organize preliminary data for the benefit of experimental and clinical researchers to develop new drug therapies by analyzing previous studies. “*Dongeuibogam*,” a representative source of the Korean medicine literature, was selected to investigate candidate antiaging medicinal herbs and to identify appropriate terms that describe the specific antiaging effects that these herbs are predicted to elicit. In addition, we aimed to review previous studies that referenced the selected candidate antiaging medicinal herbs. From our chosen source, “*Dongeuibogam*,” we were able to screen 102 terms describing antiaging effects, which were further classified into 11 subtypes. Ninety-seven candidate antiaging medicinal herbs were selected using the criterion that their antiaging effects were described using the same terms as those employed in “*Dongeuibogam*.” These candidates were classified into 11 subtypes. Of the 97 candidate antiaging medicinal herbs selected, 47 are widely used by Korean medical doctors in Korea and were selected for further analysis of their antiaging effects. Overall, we found an average of 7.7 previous studies per candidate herb that described their antiaging effects.

## 1. Introduction

Recently, a number of studies have been conducted that pursue the active development of antiaging drugs. Many researchers develop novel drugs by exploring the antiaging constituents of herbs that are widely used in traditional medicine in many countries around the world. For example, in previous studies, preliminary data were identified by searching for candidate herbs in the traditional medicinal literature and then evaluating the antiaging effects of these candidates (e.g., [1, 2]). However, thus far, such studies have been conducted only within the traditional Chinese literature, while the Korean literature remains to be analyzed.

Therefore, we have reviewed a representative source of classical Korean medical literature as a means of providing useful preliminary data, as has been done previously with the Chinese literature. There are several justifications for selecting “*Dongeuibogam*,” which was published in 1613, for analysis in the present study; (1) it was published by the royal physicians, who were contemporary experts that strictly upheld the traditions of basic Korean medicine (KM); from ancient to present times, KM has been developed via exchange with adjacent countries such as China and Japan. Recently, this traditional medicinal science has contributed to academic development as well as to the improvement of human health through exchange with Western medicine; (2) “*Dongeuibogam*” is the comprehensive summary of all the traditional medicines of North-East Asia prior to the 17th century, because it is based on a rigorous selection of 189 of the major medicinal literature sources of the region [3]; (3) it had a significant impact not only on KM after the 17th century but also on medicinal practices in other surrounding countries (e.g., China and Japan) [4]; (4) except for minor content related to superstitions, which were contemporary standards at the time of publication, most of its content is still widely used in modern KM by Korean medical doctors (KMDs); and (5) the medicinal herbs which it describes constitute many of the major herbs prescribed in KM [5]. Taken together, it seems reasonable to conclude that “*Dongeuibogam*” is a principal piece of KM literature and summarizes all the achievements of traditional KM. Therefore, we determined that analyzing, screening, and organizing terms describing antiaging effects (TAE) in the “*Dongeuibogam*” is an efficient approach for creating lists of candidate antiaging medicinal herbs (CAMH). Furthermore, pursuing this approach may help to organize preliminary data for future experimental and clinical studies on the antiaging effects of previously investigated medicinal herbs.

## 2. Materials and Methods

The present study consisted of three steps. In the first step, TAEs were screened to construct lists of TAEs. In the second step, CAMHs were screened to construct lists of CAMHs. In the last step, previous studies of CAMHs were analyzed. TAEs and CAMHs were defined by analyzing various sources of the Northeast Asian medicinal literature; TAEs in “*Dongeuibogam*” were defined as terms describing potency for delaying/improving specific aging symptoms that are recognized by one's human sense or others. In contrast, CAMHs were defined as medicinal herbs containing one or more TAEs in their medicinal potency [6]. Each step was performed as described in the following paragraphs.

### 2.1. First Step (Figure 1): Screening TAEs Found in the “Dongeuibogam” and Establishing a List of TAEs

#### 2.1.1. Selection of 928 Individual Medicinal Herbs (IMH) in the “Dongeuibogam”

Although there were 1,932 IMHs listed in the “*Dongeuibogam,*” overlapping items were excluded and 928 IMHs were ultimately identified. IMH files were selected if commercially accessible; selected items were revised per* A New Enlarged Edition*:* A Translation Printed Side by Side with Original Dongeuibogam *(Research team for classical Korean medical literature, 2012) prior to use.

#### 2.1.2. Interpretation of 3,808 TAEs and Establishment of a List of Candidate TAEs

In order to meticulously interpret the TAEs of IMHs, TAEs were divided into simple descriptive units and 3,808 TAEs were identified. Each TAE was analyzed if it was related to antiaging concepts in modern science. As a result, 104 TAEs were selected as candidates. Overlapping and similar TAEs were combined, which resulted in a list of 11 subtypes.

#### 2.1.3. Expert Survey and Establishment of the List of TAEs

Twenty-six experts were recruited for the present study. These experts were faculty members of formal institutes of KM and, concomitantly, were members of the Society of Korean Medical Classics, which consists of experts of the classical medical literature. We distributed questionnaires related to the lists of candidates and the selection criteria for TAEs in order to collect expert suggestions (period: 07.24.2014–08.10.2014). In the end, a TAE was selected only if more than 50% of respondents chose it and, hence, our data were narrowed to a list of 102 TAEs. The justification for setting the criteria at 50% was to retain a wider range of TAEs. The questionnaire was written by the authors and then finalized by an advisory panel consisting of basic KM researchers, clinical KM researchers, and biological science researchers (*n* = 6).

### 2.2. Second Step (Figure 2): Selection of CAMHs from the “Dongeuibogam” and Establishment of the List of CAMHs

#### 2.2.1. Selection of Preliminary 97 CAMHs

Utilizing TAEs from the first step, 97 CAMHs were selected, which contained at least one of the TAEs.

#### 2.2.2. Establishment of a List of 94 CAMHs

Preliminary candidates from the first selection (i.e., 97 CAMHs) were filtered into 94 CAMHs. Two were excluded because they were not from a single source and another was filtered because it was clearly based on superstition. Following this exclusion, the final list of CAMHs was established.

### 2.3. Third Step (Figure 3): Preliminary Evaluation of the Antiaging Effects of CAMHs via Analysis of Previous Studies

#### 2.3.1. Selection of Medicinal Herbs for Preliminary Evaluation of the Candidate Lists

Of the 94 CAMHs, the authors included medicinal herbs that are commonly used by KMDs. Following discussion with the advisory panel, 47 candidates were selected (i.e., 43 plant-derived and 4 animal-derived) for further preliminary evaluation. Ginseng Radix and honey were excluded despite their common use as medicinal ingredients because of an excessively broad range of applications.

#### 2.3.2. Selection and Analysis of Previous Studies regarding Antiaging Effects

We searched for 47 different medicinal herbs in the previous studies and identified relevant studies concerning several major hypotheses of aging (e.g., the free radical theory [319], oxidative stress theory [320], molecular inflammation hypothesis [321, 322], neuroendocrine theory [323], wear and tear theory [324], waste accumulation theory [325], Hayflick limit theory [326], and the telomerase theory [327]). Additional studies were included after discussion with the advisory panel. Next, studies were specifically divided into* in vitro* studies,* in vivo* studies, clinical studies, and reviews, and then analyzed again for research performance status.

#### 2.3.3. Searching the Database

In addition to commonly used scientific databases (such as PubMed, Cochrane, and Scopus), Korean databases (Ndsl, Oasis, and Riss) were used since we were searching specifically for studies related to KM. The starting period for these study searches was not defined; however, July 31, 2014 was set as the final time point.

#### 2.3.4. Searching Keywords

We used the following terms for the searches: “scientific names of CAMH + aging, age” and “names of herbal medicines of CAMH + aging, age.”

## 3. Results and Discussion

### 3.1. List of TAEs from the “Dongeuibogam”

The TAEs of 928 IMHs in the* “Dongeuibogam”* were divided by simple descriptive units to achieve 3,808 TAEs. In the first step, TAEs for disease treatments were excluded, resulting in 593 TAEs. Of this subset, overlapping TAEs were combined into a singular TAE list containing 333 TAEs. In the second step, 299 TAEs were excluded as they described general health. Thus, 104 TAEs specifically related to aging were selected. In order to validate the above processes, we consulted a survey of experts. Ten out of 11 respondents agreed with the validity of the first step, while one respondent disagreed (90.9% versus 9.1%). With regard to the validity of the second step, 8 out of 10 respondents agreed (80% versus 20%) (Table 1).

TAEs selected through the processes described above were further divided into 11 types of lists: 21 skin-related TAEs, 15 hair-related TAEs, 15 musculoskeletal TAEs, 14 sensory organ-related TAEs, 12 TAEs related to the extension of life span, 13 cognitive function-related TAEs, 5 tooth-related TAEs, 5 sexual function-related TAEs, 2 urination-related TAEs, 1 oral health-related TAE, and 1 respiratory function-related TAE. Classified TAEs were further assessed for proper categorization via questionnaires. In the end, depending upon the TAE, the agreement ratio for validation of each type ranged from 38.5% to 100%. Two TAEs were excluded as these lists had less than 50% agreement on the validation and, therefore, 102 TAEs were finalized for further analysis. In order to avoid overlapping TAE lists, they are summarized with the lists of CAMHs as follows. 

*Total of 102 TAEs and 94 CAMHs Divided into 11 Subtypes (Some Items Were Medicinal Herbs Containing One or More TAEs and Were Excluded)*

*TAE/The Number of Respondents for “Validity” = N*(%)*/CAMHs*

*(i) Skin-Related: 21 TAEs and 22 CAMHs*

 Skin becomes glossy/9 (69.2%)/Ginseng Radix
 Removes the wrinkles even from an old man/13 (100%)/Endocarpium Castaneae Mollissimae
 Adds sheen to the face of the age/12 (92.3%)/Suis Unguis
 Adds sheen to the face/10 (76.9%)/Leonuri Herba
 Adds sheen to the face/10 (76.9%)/Benincasae Semen
 Adds sheen to the face/10 (76.9%)/Batryticatus Bombyx
 Adds sheen to the face/10 (76.9%)/Lithar Gyrum
 Improves complexion/10 (76.9%)/Leonuri Herba
 Adds sheen to the face/10 (76.9%)/Margaritum
 Removes wrinkles/12 (92.3%)/Cervi Cornu
 Fattens, whitens, and brightens the person/9 (69.2%)/Human milk
 Improves facial complexion/9 (69.2%)/Rubi Fructus
 Restores luster to a person/10 (76.9%)/Schisandrae Fructus
 Makes the facial skin smoother/10 (76.9%)/Cervi Cornu
 Makes the face look young/12 (92.3%)/Poria Sclerotium
 Adds smoothness to the face/11 (84.6%)/Honey
 Adds sheen to the face/10 (76.9%)/Trichosanthis Radix, Persicae Flos, and AdepsSelenarcti et Ursi
 Removes wrinkles on the hands and face/12 (92.3%)/Trichosanthis Radix
 Adds sheen to the face/10 (76.9%)/Ligustici Tenuissimi Rhizoma et Radix and Angelicae Dahuricae Radix
 Makes the face younger/13 (100%)/Atractylodis Rhizoma (Atractylodis Rhizoma Alba) and Polygonati Rhizoma
 Improves facial complexion/9 (69.2%)/PersicaeFlos, Benincasae Semen, Rubi Fructus, Cinnabaris, and Margaritum.

*(ii) Hair-Related: 15 TAEs and 12 CAMHs*

 Reinforces the teeth and the hair/10 (76.9%)/Zanthoxyli Pericarpium
 The hair becomes black again/11 (84.6%)/Sasemi Semen
 The beard and hair do not become white/11 (84.6%)/Sophorae Fructus
 The beard will turn black/11 (84.6%)/Oil of the Juglandis Semen and Root of Musa basjooSieb. et Zucc
 The white hair will turn black again/11 (84.6%)/Siegesbeckia Herba
 Blackens the hair/11 (84.6%)/Mori Fructus and Ecliptae Herba
 Changes the white hair to black/11 (84.6%)/Mori Fructus
 The hair will become longer/10 (76.9%)/Adeps Selenarcti et Ursi
 Makes the hair and beard black, glossy and shiny/12 (92.3%)/Oil of the Juglandis Semen
 The white beard will be dyed black/12 (92.3%)/Juglandis Semen
 Makes the hair and beard grow and changes white hair to black/13 (100%)/Ecliptae Herba
 Makes the hair grow/8 (61.5%)/Root of Musa basjoo Sieb. et Zucc and Sasemi Semen
 Makes the hair grow and become black/13 (100%)/Adeps Selenarcti et Ursi
 Prevents the hair from becoming white/12 (92.3%)/Achyranthis Radix
 Blackens the hair/12 (92.3%)/Sophorae Fructus, Rehmanniae Radix (Rehmanniae Radix Crudus, Rehmanniae Radix Preparata), and Polygoni Multiflori Radix.

*(iii) Musculoskeletal-Related: 14 TAEs and 23 CAMHs*

 Cures the weakness of legs/5 (38.5%)/exclusion
 Strengthens the bones/9 (69.2%)/Magnetitum
 Strengthens the muscles and bones/9 (69.2%)/Acanthopanacis Cortex
 Makes the body feel light/11 (84.6%)/ChrysanthemiFlos, Euryales Semen, Lycii Fructus, Colophonum, Nelumbinis Semen, Acanthopanacis Cortex, Rehmanniae Radix (Rehmanniae Radix Crudus, Rehmanniae Radix Preparata), Acori Gramineri Rhizoma, Asparagi Tuber, Atractylodis Rhizoma (Atractylodis Rhizoma Alba), Cuscutae Semen, Pini Koraiensis Semen, Sasemi Semen, Polygonati Rhizoma
 Strengthens the muscles and bones/9 (69.2%)/Siegesbeckia Herba
 Strengthens the muscles and bones/9 (69.2%)/Chaenomelis Fructus
 Strengthens the muscles and bones/9 (69.2%)/Eucommiae Cortex
 Strengthens the muscles/8 (61.5%)/Animalis Nervus; exclusion
 Strengthens the power/7 (53.8%)/Rubi Fructus
 Strengthens the muscles/7 (53.8%)/Chaenomelis Fructus
 Helps one gain power/7 (53.8%)/Epimedii Herba, Rehmanniae Radix (Rehmanniae Radix Crudus, Rehmanniae Radix Preparata)
 Gives strength/7 (53.8%)/Rubi Fructus
 Strengthens the muscles and bones/8 (61.5%)/Chrysanthemi Flos, Cervi Parvum Cornu, Schisandrae Fructus
 Strengthens the muscles/7 (53.8%)/Polygoni Multiflori Radix
 Treats a general lack of enthusiasm/7 (53.8%)/Lycii Fructus.

*(iv) Related to the 5 Sensory Organs: 14 TAEs and 47 CAMHs*

 Enhances eyesight/9 (69.2%)/Brassica Rapae Radix Seu Folium, Natrii Chloridum, Galla Rhois, Naemorhedi Jecur
 Enhances eyesight and hearing abilities/10 (76.9%)/Acori Gramineri Rhizoma
 Enhances eyesight/9 (69.2%)/Chrysanthemi Flos, Canitis Fel, Cassiae Leaves, Cassiae Semen, Azuritum, Sophorae Fructus, Malachitum, Brassica Rapae Radix Seu Folium, Equiseti Herba, Rubi Fructus, Serpentis Periostracum, Haliotidis Concha, Asiasari Radix et Rhizoma, NatriiChloridum, GallaRhois, BovisJecur, Human milk, Suis Testis, Gapsellae Bursa-pastoris Semen, Citrus Unshius Pericarpium, Xanthii Fructus, Naemorhedi Jecur, Sal, AtractylodisRhizoma AtractylodisRhizoma Alba, LepiJecur, Cuscutae Semen, Phellodendri Cortex, and Coptidis Rhizoma
 Enhances eyesight and cures weak vision/9 (69.2%)/Sophorae Fructus, Vespertilii Excrementum, and Naemorhedi Jecur
 Treats blurred vision/9 (69.2%)/Viticis Fructus
 Treats blurred vision/9 (69.2%)/Cicadae Periostracum
 Treats blurred vision/9 (69.2%)/Galli Mas Os Nigri Fel
 The vision is unclear/7 (53.8%)/Lutrae Fel
 Brighten the eyes/9 (69.2%)/Siegesbeckia Herba
 Enhances the vision/9 (69.2%)/Citrus Unshius Pericarpium
 Improves the eyesight/9 (69.2%)/Serpentis Periostracum and Human milk
 Cures the weak vision/9 (69.2%)/Mirabilitum
 Cures the weak vision/9 (69.2%)/Lepi Jecur
 Makes the hearing and the vision better/10 (76.9%)/Euryales Semen.

*(v) Related to Extension of Life Span: 12 TAEs and 37 CAMHs*

 Lengthens the life/13 (100%)/Poria Sclerotium
 Keeps one young/13 (100%)/Chrysanthemi Flos, Euryales Semen, Poria Sclerotium, Nelumbinis Semen, and Acanthopanacis Cortex
 Elongates the life/13 (100%)/Atractylodis Rhizoma (Atractylodis Rhizoma Alba)
 Keeps one young/12 (92.3%)/Euryales Semen
 Keeps one young/13 (100%)/Cervi Parvum Cornu
 Ensures a long life/12 (92.3%)/Nelumbinis Semen
 Ensures a long life/12 (92.3%)/Lycii Fructus
 Keeps one young/13 (100%)/Cervi Parvum Cornu, Mori Fructus, Colophonum, Rehmanniae Radix (Rehmanniae Radix Crudus, Rehmanniae Radix Preparata), Acori Gramineri Rhizoma, Atractylodis Rhizoma (Atractylodis Rhizoma Alba), Polygoni Multiflori Radix, Pini Koraiensis Semen, Sasemi Semen, and Polygonati Rhizoma
 Elongates the life/12 (92.3%)/Thujae Orientalis Folium, Poria Sclerotium, Colophonum, Nelumbinis Semen, Acanthopanacis Cortex, Acori Gramineri Rhizoma, Asparagi Tuber, Atractylodis Rhizoma (Atractylodis Rhizoma Alba), Cuscutae Semen, Polygoni Multiflori Radix, Pini Koraiensis Semen, and Sasemi Semen
 Elongates the life/13 (100%)/Human milk
 Elongates the life/13 (100%)/Chrysanthemi Flos, Thujae Orientalis Folium, Acori Gramineri Rhizoma, Atractylodis Rhizoma (Atractylodis Rhizoma Alba), and Polygoni Multiflori Radix
 Ensures a long life/12 (92.3%)/Euryales Semen, Sophorae Fructus, Atractylodis Rhizoma (Atractylodis Rhizoma Alba), Sasemi Semen, and Multae Flores; exclusion.

*(vi) Cognitive Functions-Related: 12 TAEs and 9 CAMHs*

 Cures forgetfulness/10 (76.9%)/Calculus, Polygalae Radix, Hominis Placenta, Suis Cordis, and Aranea Ventricosa Cobwe (exclusion)
 Cures forgetfulness/10 (76.9%)/Ginseng Radix
 Makes one smart/9 (69.2%)/Acori Gramineri Rhizoma
 Cures forgetfulness/10 (76.9%)/Hoelen cum Radix
 Cures forgetfulness/10 (76.9%)/Aranea Ventricosa Cobwe; exclusion
 Nurtures the spirit/9 (69.2%)/Nelumbinis Semen
 Makes one smart/9 (69.2%)/Polygalae Radix, Alpiniae Oxyphyllae Fructus, and Ginseng Radix
 Makes one smart/9 (69.2%)/Acori Gramineri Rhizoma
 Makes one smart/9 (69.2%)/Polygalae Radix
 Makes one smart/8 (61.5%)/Acori Gramineri Rhizoma
 Cures forgetfulness/10 (76.9%)/Calculus
 Makes one's mind feel cool/8 (61.5%)/Nelumbinis Semen
 Cools the head and eyes/6 (46.2%)/exclusion.

*(vii) Tooth–Related: 5 TAEs and 9 CAMHs*

 Reinforces the teeth and the hair/11 (84.6%)/Zanthoxyli Pericarpium
 Reinforces the teeth/9 (69.2%)/Bovis Dens
 Reinforces the teeth/9 (69.2%)/Drynariae Rhizoma, Sophorae Fructus, Cervi Parvum Cornu, Natrii Chloridum, and Tribuli Fructus
 Strengthens the teeth/9 (69.2%)/Ashes of a sheep's Tibia, Sal
 Stimulates the growth of teeth/8 (61.5%)/Cervi Parvum Cornu.

*(viii) Related to Sexual Functions: 5 TAEs and 7 CAMHs*

 Strengthens the sexual function/7 (53.8%)/Passeris Caro
 Cures the impotence/9 (69.2%)/Bombyxmori L.
 Strengthens the sexual function/8 (61.5%)/Passeris Caro
 Cures the impotence/9 (69.2%)/Canitis Penis et Testis, Rubi Fructus, Otariae Testis et Penis, Achyranthis Radix, Epimedii Herba
 Cures the impotence/9 (69.2%)/Canitis Penis et Testis.

The following 4 TAEs are for disease treatment. But they were selected as TAEs because they have the words, “the elderly.”

*(ix) Urination-Related: 2 TAEs and 2 CAMHs*

 Cures the abnormal urination of the elderly/8 (61.5%)/Corni Fructus
 Cures the enuresis of the elderly/8 (61.5%)/Achyranthis Radix.

*(x) Oral Health-Related: 1 TAE and 1 CAMH*

 Cures the canker sore of the elderly/8 (61.5%)/Human milk.

*(xi) Respiratory Function-Related: 1 TAE and 1 CAMH*

 Cures the chronic cough of the elderly/8 (61.5%)/Armeniacae Semen.

### 3.2. Lists of CAMHs in the “Dongeuibogam”

Ninety-seven medicinal herbs associated with at least one TAE were selected from the “*Dongeuibogam.*” Among these herbs, three items were excluded because “Multae Flores” and “Animalis Nervus” stand for various flowers and multiple animals' muscles, respectively. In addition, the unreasonable item, “Aranea ventricosa cobweb,” was also excluded. Therefore, 94 CAMHs were finally selected. These candidates were divided into categories for either internal or external use, and were then subdivided into plant-derived, animal-derived, and mineral-derived medicinal herbs as follows.

*CAMHs were Divided into Categories for Either Internal or External Use, Subdivided into Plant-Derived, Animal-Derived, and Mineral-Derived Medicinal Herbs*

*(i) 69 CAMHs for Internal Use*

*45 Plant-Derived Medicinal Herbs. *Acanthopanacis Cortex/Achyranthis Radix/Acori Gramineri Rhizoma/Alpiniae Oxyphyllae Fructus/Armeniacae Semen/Asiasari Radix et Rhizoma/Asparagi Tuber/Atractylodis Rhizoma (Atractylodis Rhizoma Alba)/Brassica Rapae Radix Seu Folium/Cassiae Leaves/Chaenomelis Fructus/Chestnut/Chrysanthemi Flos/Citrus Unshius Pericarpium/Colophonum/Coptidis Rhizoma/Corni Fructus/Cuscutae Semen/Ecliptae Herba/Epimedii Herba/Equiseti Herba/Eucommiae Cortex/Euryales Semen/Gapsellae Bursa-pastoris Semen/Ginseng Radix/Hoelen cum Radix/Lycii Fructus/Mori Fructus/Nelumbinis Semen/Persicae Flos/Phellodendri Cortex/Pini Koraiensis Semen/Polygalae Radix/Polygonati Rhizoma/Polygoni Multiflori Radix/Poria Sclerotium/Rehmanniae Radix (Rehmanniae Radix Crudus, Rehmanniae Radix Preparata)/Rubi Fructus/Sasemi Semen/Schisandrae Fructus/Siegesbeckia Herba/Sophorae Fructus/Thujae Orientalis Folium/Viticis Fructus/Xanthii Fructus.

*20 Animal-Derived Medicinal Herbs*. Bombyx mori L./Bovis Calculus/Bovis Jecur/Canitis Fel/Canitis Penis et Testis/Cervi Cornu/Cervi Parvum Cornu/Cicadae Periostracum/Haliotidis Concha/Hominis Placenta/Honey/Human milk/Lepi Jecur/Naemorhedi Jecur/Otariae Testis et Penis/Passeris Caro/Serpentis Periostracum/Suis Cordis/Suis Testis/Vespertilii Excrementum. 

*4 Mineral-Derived Medicinal Herbs*. Azuritum/Cinnabaris/Magnetitum/Malachitum.

*(ii) 25 CAMHs for External Use*

*13 Plant-Derived Medicinal Herbs*. Angelicae Dahuricae Radix/Benincasae Semen/Cassiae Semen/Drynariae Rhizoma/Endocarpium Castaneae Mollissimae/Galla Rhois/Juglandis Semen (Oil of the Juglandis Semen)/Leonuri Herba/Ligustici Tenuissimi Rhizoma et Radix/Root of Musa basjoo Sieb. et Zucc/Tribuli Fructus/Trichosanthis Radix/Zanthoxyli Pericarpium.

*7 Animal-Derived Medicinal Herbs*. Adeps Selenarcti et Ursi/Batryticatus Bombyx/Bovis Dens/Galli Mas Os Nigri Fel/Lutrae Fel/Suis Unguis/Tibia of a sheep's ashes.

*5 Mineral-Derived Medicinal Herbs*. Lithar Gyrum/Margaritum/Natrii Chloridum/Natrii Sulfas/Sal.

Lastly, these were classified utilizing TAEs as well (since there were medicinal herbs possessing one or more TAEs, some items overlapped; as explained previously).

### 3.3. Preliminary Evaluation of the Antiaging Effects of CAMHs via Analysis of Previous Studies

Through discussion with advisory panels, the authors selected 47 kinds of CAMHs (i.e., 43 plant-derived kinds and 4 animal-derived kinds) that are commercially available and widely utilized by KMDs. A total of 3,146 studies of 47 CAMHs were found; of these, 363 studies were concerned with antiaging effects, resulting in an average of 7.7 publications per candidate herb (Table 2).

As depicted in Table 2, 43 kinds of CAMHs were studied and their antiaging activity was corroborated by more than one research study (except Equiseti Herba, Gapsellae Bursa-pastoris Semen, Poria Sclerotium, Siegesbeckia Herba, Sophorae Fructus, and Viticis Fructus). Among these publications, there were medicinal herbs assessed in multiple studies with various references to their potency against aging. For instance, there were 58 publications found for Lycii Fructus, 25 for Epimedii Herba, 24 for Polygoni Multiflori Radix, and 23 for Mori Fructus. In contrast, only one relevant study each was found for Euryales Semen, Thujae Orientalis Folium, and Xanthii Fructus. However, regardless of the number of previous studies, the finalized list of CAMHs should be investigated for their antiaging potency because these CAMHs were carefully selected by TAE criteria that were agreed upon by the consultation and agreement of experts.

Since the present study was performed with a focus on the selection and cataloging of an entire candidate group of antiaging medicinal herbs written about in the* “Dongeuibogam*,” the characteristics of each medicinal herb were not analyzed in detail during both the discovery processes from the classical Korean medical literature and the analysis processes of preceding studies. This constitutes a limitation of the present study but is also an advantage because the scope of this study is comprehensive. This part will be included in a follow-up study on the verification of the antiaging effects of each CAMH.

Furthermore, additional investigation is warranted for the “Compound formulae” (mixture of medicinal herbs) identified in “*Dongeuibogam*” as an expansion of the present study that limited putative candidates to IMHs.

## 4. Conclusions

In the present study, we finally selected 47 CAMHs from the “*Dongeuibogam*” and reviewed the results of previous studies regarding antiaging effects in order to provide a comprehensive list of Korean medicinal herbs that may harbor antiaging potential. Even though further investigations are needed in regard to the medicinal herbs included in these lists, the present study may be an important step towards the development of experimental and clinical studies with the aim of discovering new drugs or novel antiaging constituents.

## Figures and Tables

**Figure 1:**
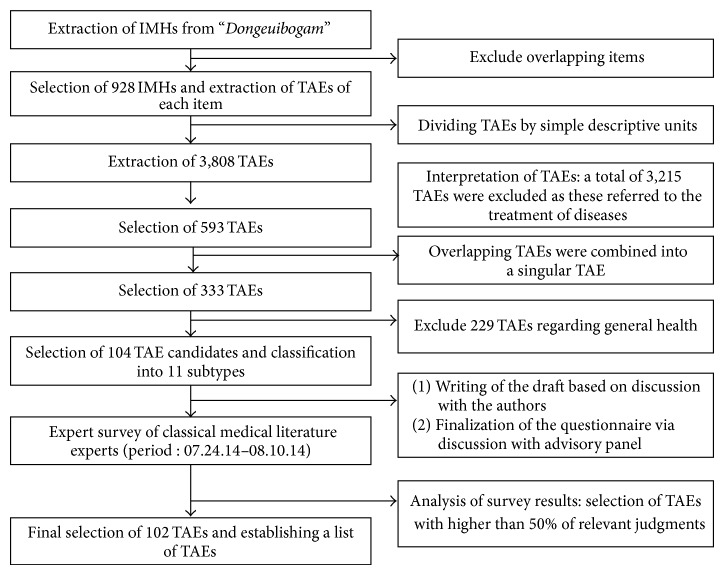
1st research process. Screening TAEs found in the “Dongeuibogam” and establishing a list of TAEs.

**Figure 2:**
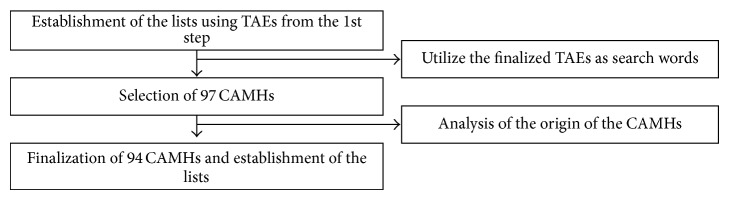
2nd research process. Selection of CAMHs from the “Dongeuibogam” and establishment of the list of CAMHs.

**Figure 3:**
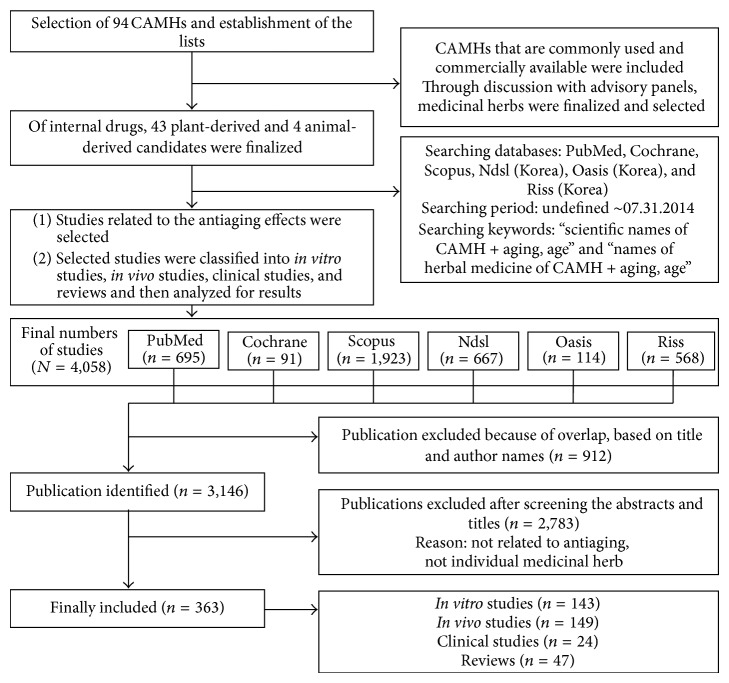
3rd research process. Preliminary evaluation of the antiaging effects of CAMHs via analysis of previous studies.

**Table 1:** Results of survey regarding study methods.

Question	The number of respondents for “validity” = *N* (%)	The number of respondents for “no validity” = *N* (%)	Total respondents/total subjects = *N* (%)
1	*N* = 10 (90.9%)	*N* = 1 (9.1%)	*N* = 11/26 (42.3%)

2	*N* = 8 (80%)	*N* = 2 (20%)	*N* = 8/26 (38.5%)

**Table 2:** Preliminary evaluation of the antiaging effects of 47 CAMHs via analysis of previous studies.

Name of CAMH/classification of the study (number)/source database/main outcome		
Plant-derived medicinal herbs		
Acanthopanacis Cortex	VT (2)	(1) S, R, N/suppression of oxidative DNA damage in lymphocytes [7]
		(2) R/promotion of *B. adolescentis* (probiotics) [8]

Achyranthis Radix	VT (3)	(1) S/protects oxidative damage to DNA [9]
		(2) S/increases DPPH radical scavenging activity [10]
		(3) P, S/increase activity of SOD and GSH-Px [11]
	VV (11)	(1) P, S/increase lymphocyte proliferation activity [12]
		(2) R/decreases WBC counts in mice with induced arthritis [13]
		(3) P, S/improve the actions of enhancing memory and endurance [14]
		(4) S/increases expression of BMP-2 in bone tissue on albumen levels [15]
		(5) S/improves activity of SOD [16]
		(6) P, S, N/nonenzyme glycation in D-galactose [17]
		(7) P, S/restore deficiency of the immune system [18]
		(8) R/prevents autoimmune inflammatory joint diseases (CD56, IL-1*β*) [19]
		(9) O/reduces serum levels of TNF-*α* and PGE2 collagen-induced arthritis [20]
		(10) O/decreases CRP and WBC levels in induced arthritis [21]
		(11) R/decreases WBC counts in induced arthritis [22]
	C (1)	(1) C/improves osteoarthritis [23]
	R (3)	(1) S/anti-inflammatory plant saponins [24]
		(2) S/decreases bone loss in OVX rats by inhibiting osteoclast formation [25]
		(3) R/improves osteoporosis [26]

Acori Gramineri Rhizoma	VT (3)	(1) R/promotes *C. butyricum* (probiotics) [8]
		(2) P, S, N/absence of inhibitory activities on AChE [27]
		(3) R, N/repair the degeneration of neuroblastoma cells by CT105 expression [28]
	VV (1)	(1) P, S, R/improve cognitive function in aged animals [29]
	R (1)	(1) S/alleviates age-related dementia and memory impairment [2]

Alpiniae Oxyphyllae Fructus	VT (1)	(1) P, S/antioxidant and free radical scavenging potential of yakuchinone B derivatives [30]
	VV (2)	(1) S, N/alpinia protocatechuic acid protects against oxidative damage and stress [31]
		(2) P, S, N/elevated splenic weights, increased activities of GSH-PX, and CAT [32]

Armeniacae Semen	VT (2)	(1) S/increases total antioxidant activity (TAA) [33]
		(2) P/increases *in vitro* activity of almond skin polyphenols for scavenging free radicals and induces quinone reductase [34]
	VV (5)	(1) P/increases bone mass [35]
		(2) P/increases activities of SOD, GSH-Px, and the liquid fluidity of the erythrocyte membrane [36]
		(3) P/increases the lag time to LDL oxidation [37]
		(4) P/enhances rat working memory in the Morris water maze [38]
		(5) P/downregulates the levels of proinflammatory cytokines (interleukin [IL]-1*β*, tumor necrosis factor-*α*, and interferon-*γ*) and upregulates the levels of anti-inflammatory cytokines (IL-4, IL-2, and IL-10) [39]
	C (3)	(1) S/increases erythrocyte SOD activity [40]
		(2) P/resists oxidative damage (changes in plasma F(2)-isoprostane levels) [41]
		(3) P/C/restore solar skin fluorescence [42]

Asiasari Radix et Rhizoma	VT (1)	(1) S/exhibits DPPH radical scavenging activity [43]

Asparagi Tuber	VT (1)	(1) S/stimulates osteoblast differentiation and inhibits osteoclast generation [44]
	VV (1)	(1) P, S/increased the spleen index and the SOD activity with polysaccharides and aqueous extracts of the roots [45]
	R (1)	(1) S/reduces age-related dementia and memory impairment [2]

Atractylodis Rhizoma (Atractylodis Rhizoma Alba)	VV (3)	(1) S, N/reduce the levels of MDA, Lipo, and the activity of MAO in serum and brain tissue [46]
		(2) O/improves arthritis score [47]
	C (2)	(1) N/reduces Crow's feet area (antiwrinkle for eye) [48]
		(2) N/reduces Crow's feet area (antiwrinkle for eye) [49]
	R (2)	(1) S/reduces age-related dementia and memory impairment [2]
		(2) S/reduces glaucoma [50]

Cassiae Leaves	VT (2)	(1) S/ has effect on estrogen receptor- (ER-) positive MCR-7 cell proliferation [51]
		(2) P/peroxynitrite scavenging [52]
	VV (1)	(1) S/reduces intraocular pressure [53]

Chaenomelis Fructus	VV (3)	(1) P/improves aging in cultured rat fibroblasts [54]
		(2) O/improves arthritis index (the incidence of arthritis and the degree of joint edema) [55]
		(3) R/improves the aged ovariectomized rat of postmenopausal osteoporosis [56]
	R (1)	(1) S/improves osteoarthritis [57]

Chestnut	VT (6)	(1) R/increases the scavenging effect on DPPH radicals [58]
		(2) N/protects hydrogen peroxide-induced oxidative DNA damage [59]
		(3) R/increases the scavenging effect on DPPH radicals [60]
		(4) N/inhibits elastase activity (antiwrinkle effect) [61]
		(5) N/inhibits elastase activity (antiwrinkle effect) [62]
		(6) R/inhibits tyrosinase activity [63]

Chrysanthemi Flos	VT (2)	(1) S/ has efficacy as an antioxidant and inhibits the formation of melanin and antimutagenicity [64]
		(2) R/improves antiallergic cosmeceuticals [65]
	VV (1)	(1) P, S/decrease MDA content and Ach E activity and increase SOD activity in aging mice [66]
	R (1)	(1) S/reduces glaucoma [50]

Citrus Unshius Pericarpium	VT (2)	(1) P, S/increase antioxidative and photoprotective effects of coumarins [67] (2) S/decreases MMP-1 mRNA expression [68]
	R (1)	(1) S/improves impotence [69]

Colophonum	VT (3)	(1) O/inhibits NO production and ROS generation in LPS-stimulated RAW264.7 cells [70] (2) O/reduces DPPH free radicals [71] (3) N, O/increase ROS scavenging effects [72]
	VV (4)	(1) P/inhibits Fe(2+)-induced lipid peroxidation [73]
		(2) N/increases membrane fluidity in Liver [74]
		(3) N/attenuates oxygen radicals and activates scavenger enzymes [75]
		(4) N/decreases LDL-cholesterol levels [76]
	C (1)	(1) N, O/skin-lifting effect and a decrease in corneocytes [77]

Coptidis Rhizoma	VT (1)	(1) P, S/inhibit indoleamine 2, 3-dioxygenase activity [78]
	R (2)	(1) P, S/higher activity than donepezil (computational pharmaceutical analysis) [79]
		(2) S/increases antioxidants in dementia [80]

Corni Fructus	VT (12)	(1) P, S, N/inhibit AGEs in the diabetic kidney [81]
		(2) S/increases anti-AChE activities [82]
		(3) N/increases cell activity and CDK4 mRNA content and decreases cyclin D1 mRNA content [83]
		(4) N/decreases the cell activity and increase the contents of *β*-galactosidase and the positive cell number of p16 [84]
		(5) R/increases SOD and catalase activities [85]
		(6) S/Increases antiosteoporotic effects (radical scavenging activity) [9]
		(7) S/inhibits free radicals and AGEs formation [86]
		(8) R/inhibits Corni Fructus extracts on AGEs [87]
		(9) N, R/increases radical scavenging activity in the DPPH assay [88]
		(10) O/protects the hydrogen peroxide-induced damage of HEI-OC1 auditory cells [89]
		(11) O/enhances the proliferation of cultured splenocytes and thymocytes [90]
		(12) R/promotes *B. adolescentis* (probiotics) [8]
	VV (3)	(1) S/effects of *Cornus* on NK activity, IL-2 activity, and IL-2 mRNA expression in aging mice [91]
		(2) P, S, N/protect rat mesangial cell proliferation [92]
		(3) N, R/increase *ω*-3 fatty acids (ALA, EPA, and DHA) content [93]
	R (2)	(1) S/reduces age-related dementia and memory impairment [2]
		(2) S/reduces glaucoma [50]

Cuscutae Semen	VT (2)	(1) P, S, N/protect murine osteoblastic MC3T3-E1 cells against tertiary butyl hydroperoxide-induced injury [94]
		(2) S/suppresses growth and controls gene expression in CWR22Rv1 cells (prostate cancer) [95]
	VV (4)	(1) P, S/reduce mitochondrial DNA deletions and increases the activities of mitochondrial respiratory chain complexes I and IV [96]
		(2) P, S/increase antiapoptosis effects [97]
		(3) P, S, N/increase the weights of the testes, epididymis, and pituitary gland and stimulate T and LH secretion [98]
		(4) O/protects ketoconazole-induced oxidative stress in testicular damage [99]
	C (3)	(1) S/increases serum FSH levels (menopausal symptoms treatment) [100]
		(2) S/increases bone mineral density and height in prepubescent children [101]
		(3) C/improves memory, immediate recall, recognition, dream-disturbed sleep, tinnitus, and deafness in aged people [102]
	R (2)	(1) S/screen for antiaging materia medica [103]
		(2) S/potential antiosteoporotic agent [25]

Ecliptae Herba	VT (1)	(1) S/increases antioxidant capacity (BHT, ascorbic acid, and rutin) [104]
	C (3)	(1) S/increases serum FSH levels (menopausal symptoms treatment) [100]
		(2) C, S/management of diffuse hair loss [105]
		(3) C/effects on serum C-reactive protein, tumor necrosis factor-alpha, and interleukin-6 in patients with type 2 diabetes mellitus [106]

Epimedii Herba	VT (6)	(1) P, S/delay homocysteine-induced endothelial cellular senescence involving activation of the PI3K/AKT-eNOS signaling pathway [107]
		(2) S/protects oxidative damage to DNA [9]
		(3) P, S, N/improve cognitive function for anticholinesterase activity [27]
		(4) S, P Inhibits the DPPH radical with IC_50_ values less than 10 *μ*g/mL [86]
		(5) N, R/protect oxidative damage to DNA [88]
		(6) R/development of natural antioxidants by using an improved D.O. analyzing method including simple calculation of the area under the curve [8]
	VV (14)	(1) P, S/increase SOD and GSH-Px activities and inhibits the formation of LPO and LF [108]
		(2) P, N, S/raise the hypothalamic monoamine neurotransmitter levels and improves learning and memory [109]
		(3) S/reduces the mean level of NF-*κ*B signal transduction kinase-related mRNA expression in rats' splenic lymphocytes with aging [110]
		(4) P, S/have estrogen-like and antiosteoporotic activity [111]
		(5) P, S/improve erectile function of aged rats [112]
		(6) P, R/impact the expression of transcription factors in the hypothalamus of aged rats [113]
		(7) S/improves osteoporosis [114]
		(8) R/improves estrogenic effects [115]
		(9) P, S, N/improve cognitive deficits and activates quiescent neural stem cells in aging rats [116]
		(10) S, R, N/enhance antioxidant capacities of the blood and liver [117]
		(11) S/ovariectomy-induced osteopenia [118]
		(12) R/prevents liver damage, delays aging, and makes skin white [119]
		(13) R/aggregates the decline of xenobiotic metabolizing enzyme activities [120]
		(14) R, N/long-term supplementation of EKN extract to rats from weaning to 24 months may be a burden on the liver function in old age [121]
	C (2)	(1) S/has therapeutic effects on sexual disorders and immunologic inadequacy in patients with chronic renal failure undergoing hemodialysis [122]
		(2) C/elevates estrogen levels and improves lipid metabolism in postmenopausal women [123]
	R (3)	(1) P, S/improve immunological effects [124]
		(2) S/improves osteoporosis [25]
		(3) P, S, N/effects on cell generation, survival time, immunomodulation, improvement of visceral and metabolic functions, and anti-infection [125]

Equiseti Herba		None

Eucommiae Cortex	VT (8)	(1) S/improves potent antioxidant and cytoprotective properties [126]
		(2) S/protects against UVB-induced oxidative stress and is a potential agent in the prevention of UVB-induced photoaging [127]
		(3) S/improves tissue inhibitors: TIMP-1, TIMP-2, TIMP-3, and TIMP-4 [128]
		(4) P, S, N/improve cellular defense mechanisms against UV radiation-induced photoaging [129]
		(5) S/improves radical scavenging activities and protective effects against oxidative damage [9]
		(6) R/upregulates IL-2, IL-4, GM-CSF, IFN-*γ*, and TNF-*α* genes and downregulates IL-12p70 gene [130]
		(7) R/reduces peroxynitrite [131]
		(8) R/promotes *B. infantis* (probiotics) [8]
	VV (10)	(1) S, P/curative properties for BMD and BMI in OVX rats [132]
		(2) S, P, N/improve the low turnover rate in the stratum corneum of false aged model rats [133]
		(3) S/stimulates collagen synthesis [134]
		(4) R/reduces MMP-1 mRNA expression and MMP-2 activity [135]
		(5) P/enhances the activity of SOD, GSH-Px, and NOS [136]
		(6) S, P/promote collagen synthesis [137]
		(7) S, P/promote collagen synthesis [138]
		(8) R/assists with hepatic function, prevents bone absorption and bone mineral loss [139]
		(9) R/improves osteoporosis [56]
		(10) R/enhances cell proliferation and suppresses apoptosis in the hippocampus, and alleviates age-induced memory loss (TUNEL-positive cells, caspase-3-positive cells, reduced Bax/Bcl-2 ratio, increased BrdU-positive cells) [140]
	R (2)	(1) S, P, N/improve sexual dysfunction, osteoporosis, Alzheimer's disease, aging, lupus-like syndrome, and immunoregulation [141]
		(2) P, S/wide pharmacological activities, including hepatoprotective, antitoxicant, anti-inflammatory, antioxidant, antiaging, antiosteoporosis, and neurotrophic effects [142]

Euryales Semen	VV (1)	(1) P, S/increase sSOD, CAT (except for in the kidney), and GSH-Px activities and decreases MDA content [143]

Gapsellae Bursa-pastoris Semen		None

Hoelen cum Radix	R (2)	(1) S/reduces dementia and memory impairment [2]
		(2) S/reduces glaucoma [50]

Lycii Fructus	VT (19)	(1) S/antioxidant effect of *in vitro* seeding of *Lycium barbarum* by DPPH assay [144]
		(2) N, P, S/suppress the level of lactate dehydrogenase release and the activity of caspase-3 [145]
		(3) N, P, S/enhance bioavailability of 3R,3′R-zeaxanthin dipalmitate [146]
		(4) S/protects U373 human astrocytes from hydrogen peroxide-induced cell death [126]
		(5) P, S/increase the cell viability and decreases the expression levels of P53 and P16 [147]
		(6) S, R/suppress the proteolytic activities of MMP-2/-9 [148]
		(7) P, S/protect against age-related macular degeneration [149]
		(8) S/determining the best explant and corresponding hormonal compositions for Goji *in vitro* regeneration [144]
		(9) S/regulates gene expression of c-fos [150]
		(10) S/suppress the expression of genes related to aging, such as p53, p21, and Bax [151]
		(11) N, P, S/improve neuroprotective effects on cortical neurons [152]
		(12) N, S, R/antiaging effects on human retinal pigment epithelial cells [153]
		(13) N/optimal extraction conditions for the highest activation effect on SOD activity [154]
		(14) R/used as an ethnic medicine for hypertension and promoting growth [155]
		(15) R, N/experimental study on the effect on collagen synthesis by several herbal medicines [156]
		(16) O, R/increase the scavenging activity on DPPH radicals and SOD [157]
		(17) R/promotes useful intestinal bacteria [8]
		(18) N, O/increase the scavenging activity on DPPH radicals and SOD in HS68 fibroblasts [158]
		(19) S/protects damaged hRPE cells induced by blue light irradiation [159]
	VV (25)	(1) P, S/increase SOD, CAT, and GSH-px levels in the liver [160]
		(2) P/protective effects of *Lycium barbarum* polysaccharides by Nrf2, Ho-1 antioxidant pathway [161]
		(3) N, S, R/increase SOD, decreases MDA in the serum, heart, liver, and brain [162]
		(4) S/increase serum levels of SOD and glutathione peroxidase [163, 164]
		(5) P, N, S/antiaging effects on beta-amyloid peptide neurotoxicity in Alzheimer's disease [165]
		(6) N, P, S/reduces DTT-induced LDH release and caspase-3 activity [166]
		(7) S/increases expression of the DNA repairase 8-oxoguanine gene in the liver [167]
		(8) P, S/neuroprotection by down-regulating RAGE, ET-1, A*β*, and AGE in the retina [168]
		(9) N, P, S, R/increase endogenous lipid peroxidation, and decreases antioxidant activities [169]
		(10) P, S/reduce mitochondrial DNA deletions [96]
		(11) N, R/increase learning and memory and the activities of AchE and SOD in the brain [170]
		(12) N, P, S/protect cortical neurons from Abeta_25–35_ neurotoxicity [171]
		(13) R, N/increase SOD activities and decreases MDA content [172]
		(14) P/hepatoprotective effects on CCl(4)-induced hepatic damage [173]
		(15) S/influence on the activity of SOD in the skin [174]
		(16) P, S/improve neurological deficits and reduces infarct size and cerebral edema [175]
		(17) P, S/prevent SCO-induced cognitive and memory deficits [176]
		(18) N, P, S/neuroprotective effects on transient cerebral global ischemia in gerbils [177]
		(19) N, P, S, R/neuroprotective effects of ß-amyloid peptide neurotoxicity [178]
		(20) N, P, S/neuroprotective effects on protecting retinal ganglion cells in glaucoma [179]
		(21) N, P, S/antagonizes glutamate excitotoxicity in cortical neurons [180]
		(22) N, P, S, R/downregulate the expressions of cathepsin B and cystatin C [181]
		(23) P, S/alleviate glucose metabolism disorder and reduces the generation of lipid peroxide [182]
		(24) P, S/reversal of apoptotic resistance of aged T cells by modulating the expression of apoptosis-related molecules [183]
		(25) O/increases serum HDL-cholesterol levels and collagen levels [184]
	C (3)	(1) N, P, S, C/increase fasting plasma zeaxanthin levels [185]
		(2) S/increases plasma zeaxanthin and antioxidant levels [186]
		(3) S, C/treatment effect on tinnitus [187]
	R (11)	(1) S/screening studies of traditional Chinese antiaging materia medica [103]
		(2) S/prevents kidney and liver dysfunction, visual degeneration [188]
		(3) S/Goji juice: A novel miraculous cure for longevity and well-being [189]
		(4) S/treatment of glaucoma [50]
		(5) P, S/used as or related to antiaging agents [124]
		(6) S/intercellular effects on neurons [190]
		(7) N, P, S/improve cell generation, visceral, and metabolic functions [125]
		(8) S/elaborate bioactive ingredients [191]
		(9) S/improves eyesight [192]
		(10) N, P, S, R/protect neurons against beta-amyloid peptide toxicity [193]
		(11) R/functional cosmetic with antiaging effects such as antiwrinkling, whitening, and scalp-care [194]

Mori Fructus	VT (15)	(1) N, S, P/strong scavenging activity against the hydroxyl radical (HO.) [195]
		(2) N, S, P/exhibit potent inhibitory effects on tyrosinase [43]
		(3) N/potent inhibitor of tyrosinase [196]
		(4) S/Mulberry seeds containing antioxidant polyphenolic compounds [197]
		(5) N/shows the highest elastase inhibitory activity [154]
		(6) R/shows SOD activity [198]
		(7) R/antiwrinkle effects [199]
		(8) R/increases IL-2, IL-4, IL-12p70, GM-CSF, INF-*γ*, and TNF-*α* [200]
		(9) R/DPPH radical scavenging effect [201]
		(10) R/inhibits ROS production [202]
		(11) R/Rutin, isoquercitrin, quercitrin, quercetin, (+)-dihydroquercetin, and chlorogenic acid exhibit strong antioxidant activity [197]
		(12) R/promotes *L. plantarum* (antioxidative effects) [8]
		(13) R/increases antioxidant activity of polyphenol components with DMPD assays [203]
		(14) N/inhibits tyrosinase activities [204]
		(15) R, N/contain carotene and vitamin C [205]
	VV (7)	(1) N/increases the hair regrowth effect [206]
		(2) R/reduces oxygen radicals in the liver [207]
		(3) S/exhibits a memory-enhancing effect via upregulation of NGF [171]
		(4) S/extends mean lifespan by 17% from 17.6 to 20.6 days [208]
		(5) S/shortens the duration of the telogen phase and induces the growth of anagen follicles [209]
		(6) N/decreases oxidized protein levels [210]
		(7) N/increases Cu, Zn-SOD activities in the brain cytosol [211]
	C (1)	(1) C, S/improve the MelasQOL score [212]

Nelumbinis Semen	VT (8)	(1) R/decreases DPPH radicals [58]
		(2) N, S, P/antiamnesic activity and inhibition of ChEs and BACE1 [213]
		(3) R/proliferation and apoptosis in HT-29 human colon cancer cells [214]
		(4) N, S, P/inhibit rat lens aldose reductase, AGE formation, and oxidative stress [215]
		(5)S, R, N/protect against oxidative damage to DNA [88]
		(6) R/shows potent DPPH free radical scavenging activity and potent inhibitory activity on NO production [216]
		(7) O/increases type I procollagen expression in CCD-986sk cells [217]
		(8) N, R/decrease the levels of MDA [218]
	VV (1)	(1) S/normalizes the age-associated altered levels of lipids and glucose [219]

Persicae Flos	VT (7)	(1) P/exhibits photoprotection against UVA [220]
		(2) N, R/antioxidant and hyaluronidase inhibition activities [221]
		(3) S/displays radical scavenging activity, suppresses MMP-1 expression, and increases type-1 procollagen expression [222]
		(4) S/inhibits tyrosinase [43]
		(5) P/reduces cholesterol [223]
		(6) N/shows highest antioxidant activity in methanol extracts (seeds: 98.4%) [154]
		(7) N, R/increase activities of SOD and GSH-Px and the liquid fluidity of erythrocyte membranes [224]
	VV (6)	(1) P/increases activities of SOD and GSH-Px and the liquid fluidity of erythrocyte membranes [36]
		(2) S/modulates glucose metabolism and reduces oxidative damage [225]
		(3) P/cognitive benefits [38]
		(4) P/contains proanabolic factors that can increase bone volume and restore bone [35]
		(5) S/upregulates NTH1 [226]
		(6) N/increases the basal Concentration of Extracellular Acetylcholine in the Rat [227]
	C (1)	(1) P/reduces the I/R-induced F(2)-isoprostane response [41]

Phellodendri Cortex	VT (1)	(1) N, P, S/screening to identify monoamine oxidase B inhibitors [228]
	VV (1)	(1) O/treatment effects on collagen-induced arthritis [229]

Pini Koraiensis Semen	VT (1)	(1) R/in the antioxidant activities, the results of DPPH-radical scavenging activities and SOD-like activities indicated that methanol extract had the highest activities [230]
	VV (1)	(1) R/increases liver SOD activity [231]

Polygalae Radix	VT (4)	(1) N, P, S/regulate caspase-3 and tyrosine hydroxylase [232]
		(2) N, P, S/increase the number of newly formed neurospheres [233]
		(3) R/shows high antityrosinase and antielastase activity [234]
		(4) N/inhibits apoptosis in the nervous system [235]
	VV (4)	(1) P, S/increase SOD and glutathione peroxidase [236]
		(2) N, P, S, R/increase NE and DA in the hippocampus and decreases AChE in the cortex [237]
		(3) S/increases in BDNF and its receptor TrkB expression in the hippocampal CA1 region [238]
		(4) P, S/suppress the activities of MAO and AChE [239]
	C (2)	(1) C/effects of BT-11 for enhancing cognitive functions including memory [240]
		(2) C/improves memory, immediate recall, recognition, dream-disturbed sleep, tinnitus, and deafness [102]
	R (6)	(1) S/increases cell viability and reduces cell death [241]
		(2) S/enhances age-related dementia and memory impairment [2]
		(3) S/inhibits AChE activity, improve antioxidation and enhances synaptic plasticity [242]
		(4) P/improves cognitive functions [243]
		(5) S/introduction of the Chinese Material Medica (CMM) theoretical research on Alzheimer's disease [244]
		(6) S/increases in choline acetyltransferase and NGF by Polygala tenuifolia [245]

Polygonati Rhizoma	VT (2)	(1) S/lower cytotoxicity on human epidermal melanocytes (HEMn) [43]
		(2) N, O/increase in the collagenase activity and procollagen synthesis in Hs68 human fibroblasts [246]
	VV (2)	(1) S/improves SOD activity and the cleaning activity of free radicals [247]
		(2) S/improves the activity of SOD, inhibits lipid peroxidation, and reduces the contents of MDA [248]
	R (2)	(1) N/positive effects on cell generation and improvement of visceral and metabolic functions [249]
		(2) N, R/recent advances in the study of antiaging action of Siberian Solomonseal (*Polygonatum sibiricum*) [250]

Polygoni Multiflori Radix	VT (6)	(1) P, S/inhibit staurosporine-induced apoptosis [249]
		(2) N, P, S/protect U373 human astrocytes from hydrogen peroxide-induced cell death [126]
		(3) N, O/inhibit collagenase and elastase activity [251]
		(4) N, P, S/suppresses DR4 and upregulation of Bcl-2, XIAP, and cIAP-1 [252]
		(5) S/rapid absorption of *P. multiflorum* stilbene glycoside [253]
		(6) O/protects against cell damage in UVB-irradiated HaCaT keratinocytes [254]
	VV (13)	(1) P, S/increase plasma HDL-C level and HDL-C/total cholesterol ratio and reduces plasma LPO levels [255]
		(2) S/reduces lipofuscin in the liver and brain tissues [256]
		(3) S/suppresses the destruction of Cu, Zn-SOD in the skin [257]
		(4) P, S/decrease lipofuscin percentages and MDA concentrations [258]
		(5) N, P, S/decrease hippocampal synapses count and synaptophysin expression [259]
		(6) R/improves learning-memory impairment and object recognition impairment [260]
		(7) N, P, S/neuroprotective effects through both alleviation of ERK and p38 activation [261]
		(8) N, P, S/neuroprotective effects through both alleviation of ERK and p38 activation [262]
		(9) N, P, S/decrease the spongy degeneration and lipofuscin and MDA concentrations [263]
		(10) N, S/reduce the levels of ROS, NO, and IGF-1, and increases the levels of SOD and Ca^2+^ [264]
		(11) S/strong antioxidant capacity against free radicals, lipid oxidation, and protein glycation [265]
		(12) N, R/decrease the levels of LPF in the brain and kidney and increases the activities of ATPase and SOD [266]
		(13) N, S/suppresse overexpression of *α*-synuclein [267]
	C (1)	(1) C/assessment on Mini-Mental State Examination, Ability of Daily Living Scale, and the therapeutic effect [268]
	R (4)	(1) N, S/delay the degenerative changes in the brain associated with aging [269]
		(2) S/major effects on calcium channel antagonists and antioxidant and cholinesterase inhibitors [270]
		(3) S/positive effects on cell generation and improvement of visceral and metabolic functions [125]
		(4) S/positive effects on stimulating blood circulation, supplementing vital energy, and resisting aging [271]

Poria Sclerotium		None

Rehmanniae Radix (Rehmanniae Radix Crudus, Rehmanniae Radix Preparata)	VT (2)	(1) N/biosynthesizing collagen [156]
		(2) O/antiwrinkle effects by collagenase activity and procollagen synthesis in HS68 human fibroblasts and tyrosinase activity [272]
	VV (6)	(1) P/suppresses NOX and ER stress in the brain [273]
		(2) P/protects against cholinergic and immune impairment in the mouse brain [274]
		(3) P/protects against memory damage and energy metabolism failure in aging model mice [275]
		(4) P/normalizing presynaptic proteins and their relative signaling pathways in aged rats [276]
		(5) O/antioxidative effects on kidney cell injury [277]
		(6) O/antioxidative effects on kidney cell injury [278]
	R (1)	(1) S/prevents cisplatin ototoxicity [279]

Rubi Fructus	VT (6)	(1) P, S, N/enhance osteoblast function [280]
		(2) S/regulates glycation [281]
		(3) S/improves radical scavenging activities [86]
		(4) N/inhibits elastase [282]
		(5) N/UV protection effects [283]
		(6) N/inhibits AChE and improved radical scavenging effects [284]
	VV (5)	(1) P, S, N/inhibit osteoclast differentiation induced by RANKL [285]
		(2) N, O/increase SOD and GSH-px activity of red blood cells [286]
		(3) N/increases SOD activities [287]
		(4) N/inhibits AChE activity [288]
		(5) S/suppresses age-related increases in oxidative stress [289]
	C (1)	(1) S/radical scavenging activities and protective effects against oxidative damage to DNA [9]

Sasemi Semen	R (1)	(1) S/inhibits hexanoyl dopamine formation [290]

Schisandrae Fructus	VT (7)	(1) P, S/effects on neuroprotective activity & inhibiting staurosporine-induced apoptosis [249]
		(2) P/downregulates the expression of proinflammatory genes involved in the synthesis of NO, PGE2, and TNF-*α* in lipopolysaccharide-stimulated RAW 264.7 macrophage cells by suppressing Akt-dependent NF-*κ*B activity [291]
		(3) P/inhibits the spontaneous and synchronous oscillations of intracellular Ca^2+^ through the depression of extracellular calcium influx and the initiation of action potential [292]
		(4) P/strong activity against hydroxyl radicals [195]
		(5) S, N/decrease the expression of MMP-1 protein [293]
		(6) O/protects against ROS-induced neurotoxicity [294]
		(7) O/inhibits the cell damage in UV-irradiated HaCaT cells [295]
	VV (4)	(1) P/attenuates A*β*1-42-induced memory impairment [296]
		(2) P/mitigates age-dependent impairments in mitochondrial antioxidant capacity and functional ability [297]
		(3) P/improves antibody titers against Newcastle disease virus and lymphocyte proliferation of broilers [298]
		(4) P/functions as a hormetic agent to sustain cellular redox homeostasis and mitoenergetic capacity in neuronal cells [299]
	R (1)	(1) P/stimulates liver regeneration, prevents liver injuries, and inhibits hepatocarcinogenesis as well as lipid peroxidation in rats [300]

Siegesbeckia Herba		None

Sophorae Fructus		None

Thujae Orientalis Folium	VT (1)	(1) P, S, N/decrease ROS accumulation [301]

Viticis Fructus		None

Xanthii Fructus	VT (1)	(1) O/chemopreventive potential by inhibiting the activity of cytochrome P4501A1 and free radical formation [302]

Animal-derived medicinal herbs		
Cervi Cornu	VV (1)	(1) R/increases bone mass [303]

Cervi Parvum Cornu	VT (1)	(1) O, R/improve the free radical scavenging effect [304]
	VV (5)	(1) O/inhibits immune reaction in arthritis [305]
		(2) O/inhibits DHO-DHase [306]
		(3) O/suppresses the expression of iNOS mRNA and production of NO [307]
		(4) R/increases bone mass [308]
		(5) R/prevents reductions in the bone mass and strength of the lumbar body [309]

Haliotidis Concha	VT (2)	(1) N/increases expression of type I collagen and type I procollagen [310]
		(2) N/increases expression of antioxidant enzymes such as catalase, SOD, and heme oxygenase-1 [311]

Hominis Placenta	VT (2)	(1) N/improves the radical scavenging effect [312]
		(2) N/prevents H_2_O_2_-induced apoptosis in PGT-beta cells inhibitions of iNOS and caspase-3 [313]
	VV (5)	(1) O/suppresses bone resorption by inhibition of tyrosine kinase Src, cyclooxygenase expression, and PGE2 synthesis [314]
		(2) O/suppresses type II collagen-induced arthritis [315]
		(3) O/effects on the increase of the total number of ovulated ova and the rate of morphologically normal ova [316]
		(4) O/increases the total number of ovulated ova and the rate of morphologically normal ova [317]
		(5) R/prevents osteoporosis caused by postmenopause [318]

^*^PubMed (P), Cochrane (C), Scopus (S), Ndsl (N), Oasis (O), Riss (R).

^*^ *In vitro* study (VT), *In vivo* study (VV), Clinical study (C), Review (R).

(AchE; acetylcholinesterase/AGE; advanced glycation end-product/BDNF; brain-derived neurotrophic factor/BHT; butylated hydroxy toluene/DPPH; 1,1-diphenyl-2-picrylhydrazyl/GSH; growth-stimulating hormone/HDL-C; high-density lipoprotein-cholesterol/iNOS; inducible nitric oxide synthase/MDA; malondialdehyde/MMP; matrix metalloproteinase/NO; nitric oxide/NGF; nerve growth factor/RANKL; receptor activator of nuclear factor kappa B ligand/ROS; reactive oxygen species/SOD; superoxide dismutase).
